# Alzheimer’s disease polygenic risk’s association with all-cause dementia through the plasma metabolome in the UK Biobank study

**DOI:** 10.1007/s11357-025-01724-4

**Published:** 2025-07-01

**Authors:** May A. Beydoun, Yi-Han Hu, Zhiguang Li, Michael F. Georgescu, Hind A. Beydoun, Nicole Noren Hooten, Keenan A. Walker, Mustapha Bouhrara, Lenore J. Launer, Michele K. Evans, Alan B. Zonderman

**Affiliations:** 1 Laboratory of Epidemiology and Population Sciences, National Institute on Aging, NIA/NIH/IRP, Baltimore, MD 21224 USA; 2 U.S. Department of Veterans Affairs, VA National Center On Homelessness Among Veterans, Washington, DC 20420 USA; 3 Department of Management, Policy, and Community Health, School of Public Health, University of Texas Health Science Center at Houston, Houston, TX 77030 USA; 4 Laboratory of Behavioral Neurosciences, National Institute on Aging, NIA/NIH/IRP, Baltimore, MD 21224 USA; 5 Laboratory of Clinical Investigation, National Institute on Aging, NIA/NIH/IRP, Baltimore, MD 21224 USA

**Keywords:** Alzheimer’s disease, Dementia, Polygenic risk scores, Metabolomics, Lipid metabolism, UK Biobank

## Abstract

Dementia, with Alzheimer’s Disease (AD) accounting for 60–70% of its occurrences, is a multifactorial disorder marked by cognitive and functional decline. Polygenic risk scores (PRS) stratify genetic risk for complex diseases, including AD. Integrating PRS with metabolomics offers a pathway to better understand AD etiology and identify biomarkers for early detection. We explored the association between AD PRS, metabolomics, and dementia risk using data from the UK Biobank. The analysis emphasizes sex-specific associations and the mediating role of metabolites between AD PRS and dementia risk. The study utilized data from 205,219 UK Biobank participants aged ≥ 50 years at baseline. Dementia outcomes were derived using ICD-10 codes. AD PRS were computed using genome-wide association study data, while metabolomic data included 249 biomarkers measured via Nuclear Magnetic Resonance. Time-to-event analyses (Cox proportional hazards models) and generalized structural equation modeling assessed associations and mediation effects, adjusting for age, sex, and genetic principal components. Higher AD PRS was associated with increased risks of all-cause dementia (HR: 1.75, 95% CI: 1.70–1.79, P < 0.001) and AD (HR: 2.02, 95% CI: 1.95–2.09, P < 0.001), with stronger effects in women for both outcomes when considering the main AD PRS which was related to APOE4 status. Metabolomic analyses identified lipid-related markers as key mediators. LDL phospholipid content was the metabolite marker with the strongest positive relationship with AD genetic risk (effect size, *b* = + 0.11), while HDL phospholipids percentage showed a similarly strong inverse association (effect size *b* = -0.09). No metabolomic markers were significantly associated with another version of AD PRS that was less correlated with APOE4 status. Mediation analyses applied to the main AD PRS revealed modest effects, with LDL-related metabolomic components partially mediating the effect of genetic risk on dementia incidence, through a protective type of mediation (< -2%). Nevertheless, most genetic risk operated independently of metabolites. AD PRS is significantly associated with dementia risk, with sex differences and metabolomic pathways providing further insights. Lipid metabolism, particularly LDL-centric measures, emerged as potential mediators. This integrative approach highlights the utility of combining genetic and metabolomic data to identify biomarkers and potential targets for early intervention in AD and dementia.

**Supplementary Information:**

The online version contains supplementary material available at 10.1007/s11357-025-01724-4.

## Introduction

Dementia is a multifactorial disorder characterized mainly by declining cognition, ultimately impairing daily activities [1]. Alzheimer's disease (AD) is the most common subtype, representing 60–70% of dementia cases [2]. AD is a neurodegenerative condition marked by cerebral amyloid plaques, neurofibrillary tangles, synaptic impairment, and neuronal degeneration [2, 3]. While its exact causes are unclear, genetic, environmental, and behavioral factors contribute to its onset and progression [2–5].

Polygenic risk scores (PRS) integrate the effect of multiple genetic variants to estimate an individual's genetic predisposition to complex disorders like AD [6]. By aggregating these variants, PRS can stratify individuals by genetic risk before clinical symptoms manifestation [6–8]. This approach extends beyond rare mutations in the *APP*, *PSEN1*, and *PSEN2* genes, highlighting the significance of *APOE* ε4 allele frequency in sporadic AD [6–9]. Combining PRSs with biomarkers, neuroimaging, and environmental factors may enhance personalized medicine for early AD detection, and could be used to identify individuals at high risk for later dementia, making them potential participants in studies on the onset and progression of AD [10]. However, implementation raises ethical concerns, including population-specificity and generalizability across diverse populations [6–9].

Metabolomics, a technique analyzing small molecules and metabolites, sheds light on the interplay between genetic predisposition and dementia [11]. This approach analyzes small molecules and metabolites that are involved in metabolic processes, including lipid metabolism, amino acid turnover, and oxidative stress [11]. Research has linked altered lipid profiles, reduced antioxidant capacity, and pro-inflammatory metabolites to AD, suggesting they may modify the impact of genetic susceptibility on disease outcomes [11]. Integrating AD PRS with metabolomic data can reveal key metabolic pathways, identify biomarkers for early disease detection, and inform precision medicine strategies.

This study investigates the relationship between AD PRS, the metabolome, and dementia risk. It focuses on identifying potential metabolomic mediators that link AD PRS to dementia risk, including for late-onset AD. Leveraging UK Biobank data, this research explores sex-specific associations between AD PRS and dementia/AD risk, and then uses advanced statistical tools to assess metabolomic mediation of these associations. This study aims to elucidate the molecular mechanisms underlying AD and all-cause dementia and identify metabolomic targets for intervention.

## Materials and methods

### Database

The present study uses data from the UK Biobank, a large-scale biomedical database, to track health outcomes and comorbidities among 500,000 participants aged 37–73 years at the baseline assessment (2006–2010) [12]. The dataset includes Electronic Health Records (EHR), metabolomics, genomics, and baseline assessments. EHR provides detailed information on medical histories, while metabolomics includes blood biomarkers measured using the Nightingale Health platform [12]. Genomic data is obtained from genome-wide genotyping and imputation, and polygenic risk scores are computed based on published genome-wide association studies [12]. Key baseline demographic, lifestyle, and socio-economic variables are included to account for potential confounding factors. The datasets are integrated into a unified analytical framework, allowing a multidimensional exploration of health outcomes and underlying biological mechanisms within the UK Biobank cohort. The Northwest Multi-Centre Research Ethics Committee approved the UK Biobank project, and the Institutional Review Board of the National Institutes of Health, along with the UK Biobank Access Management System, have approved the current research under application #77963.

#### Dementia outcomes

Hospital inpatient records (Hospital Episode Statistics [HES]) were used to identify dementia for all participants. A subset of participants (45%) also had primary care (General Practice [GP]) data available to ascertain dementia risk. ICD-9 and ICD-10 codes were used to determine dementia risk in the full cohort. For GP data, ICD-9 and ICD-10 were extracted from Read v2 and Clinical Terms Version 3 (CTV3) using the UK Biobank Resource 592 (Clinical coding classification systems and maps) to guide the conversion. Diagnostic codes for dementia were identified using the UK NHS National Institute for Health and Care Excellence Quality and Outcomes Framework Business Rules, version 37.0.

Dementia was identified using a designated algorithm (Refer to fields 42,018 and 42,020). Based on these variables, we removed participants whose age at dementia onset was younger than their respective baseline assessment ages [13]. ICD-10 codes F00 or G30 were employed to identify the Alzheimer's disease subtype of dementia. Nonetheless, dementia from any etiology utilized many codes (A81.0, F00, F01, F02, F03, F05, G30, G31.0, G31.1, G31.8, and I67.3), with earliest date of occurrence being established algorithmically depending on the source of information. Similarly, AD incidence was assessed by limiting ICD-10 codes to F00 or G30.

#### AD polygenic risk score

The study used a Bayesian approach to develop and apply PRS scores to meta-analyzed GWAS summary statistics. The Standard PRS set was extracted from external GWAS data, while the Enhanced PRS set combined genetic risk from external and internal sources to the UK Biobank data[14, 15]. The present study mainly used the AD PRSs as continuous variables and stratified them into tertiles as provided in the UKB showcase (field # 26,206). An alternative AD PRS was generated from raw genome imputation data in *bgen* format for replication purposes, as detailed elsewhere[16]. Both AD PRS scores were correlated and compared across levels of *APOE4* carrier status. Two genetic variants, rs429358 and rs7412, were used to directly genotype and define *APOE* haplotypes (ε2/ε3/ε4) [17]. Quality control measures were comparable to those applied to genetic principal components, excluding participants with strong kinship [17].

#### NMR metabolomics

The UK Biobank's NMR metabolomics data is a valuable resource for understanding the relationship between circulating metabolites and health outcomes like chronic diseases, aging, and mortality [18–23]. The dataset, derived from plasma samples from around 274,000 participants, includes over 250 metabolomic biomarkers across various biochemical categories. It is clinically relevant for studies on cardiovascular diseases, diabetes, cancer, and neurodegenerative disorders[18–23]. The data is standardized and quality controlled using the Nightingale Health platform. It can be merged with other UK Biobank data for integrative analyses and included lipids, specifically 14 subclasses and cholesterol levels, as well as fatty acids like omega-3, saturated, and polyunsaturated fatty acids[18–23]. It also incorporated amino acids, glycolysis metabolic processes, and inflammatory markers like glycoprotein acetyls (GlycA), a composite biomarker of inflammation[18–23].

#### Covariates

To adjust for potentially confounding factors, models were adjusted for several covariates including age, age-squared, sex and the first 20 genetic principal components (https://www.nealelab.is/uk-biobank/ukbround2announcement), which are described in greater detail in OSM 1. Other covariates including SES and household size were also included in all models and are further detailed in OSM 2.

#### Study sample selection

The UK Biobank sample in our study included 502,131 adults aged 37-73y at baseline assessment, who consented for their data to be utilized at the time of analysis. Out of this sample, those aged 50y or more at baseline, amounted to 384,471 participants. After excluding those with missing data on socio-demographics, household size, genetic principal components and AD PRS, 367,367 participants remained. Of this group, a sub-group also had metabolomic data available, resulting in a sample of 205,806 individuals. After the exclusion of prevalent dementia cases that occurred prior to baseline assessment or within the first year, the resulting sample was 205,693. Exclusion of participants with missing SES score resulted in a final sample of 205,219, with an estimated incidence of dementia within that sample of N = 5,123 after up to 15 years of follow-up, 2,261 of whom being of the AD sub-type (Figure S1).

#### Statistical methods

Stata 18.0 (StataCorp, College Station, TX) was employed for the principal elements of this research. Means and proportions were calculated for the total sample, with further stratification by sex, and comparisons based on this demographic element were conducted using various bivariate linear and multinomial logit models.

In the subsequent phase, exposure-outcome associations were evaluated utilizing a time-to-event methodology, where time was delineated as the duration from the age of study enrollment (≥ 50 years) to the age of exit. The latter refers to either the age at the time of the incident outcome or the age at censoring (due to death or the conclusion of follow-up by December 31, 2023). Kaplan–Meier survival rates were estimated with their 95% CI and compared using a log-rank test to illustrate changes in dementia-free survival probability across the tertiles of the exposure of interest, AD PRS: T1 (low AD PRS), T2 (mid AD PRS), and T3 (high AD PRS). We therefore developed Cox proportional hazards models to investigate the relationship between AD polygenic risk scores (in tertile format) and the incidence of dementia, while additionally adjusting for baseline age, squared age, sex, and the first 20 principal genetic components, an approach adopted in a previous study [7].

Third, AD PRS was subsequently used as a primary predictor in a series of multivariable-adjusted linear regression models. Each model included distinct metabolomic potential mediators (or moderators) as an alternative outcome among the 249 metabolomic markers. The model concurrently controlled for selected possible confounders. The Stata commands *parmby*, *qqval*, and *multproc* were employed, and a volcano plot was created utilizing the R 4.4.1 *ggplot* package, which distinctly illustrates p-values and effect sizes for each of the 249 equations, including those that met the Bonferroni criteria for multiple testing adjustment (Type I error reduced to 0.05/249).

Fourth, each plasma metabolite was incorporated into a generalized structural equations model to assess the total effect of AD PRS on dementia incidence, considering various metabolomic principal components as well as individual metabolites as potential mediators. The final equation was a Weibull parametric regression model. All models incorporated all potentially confounding covariates. Tabular data and supplementary datasheets for individual metabolites were employed displaying the extent of mediation (% total effect mediated) and direction (causal or protective mediation). Models were also carried on AD incidence as a secondary analysis. A similar approach was applied to a newly developed AD PRS based on a 2022 GWAS in relation to all-cause dementia [7]. Information pertaining to the data processing employed to derive this updated version of AD PRS is provided in OSM 1. The primary and secondary analyses were compared, and each PRS was also correlated to with APOE4 carrier status, as was done in a smaller sample with proteomic data, based on a previous study [7]. In order to detect whether there was additional interactions between exposures and mediators, a four-way decomposition analysis was carried out with the metabolomic PCs and each of the AD PRS scores in relation to dementia risk, after splitting the sample into 10 equally sized sample allowing for assessment of heterogeneity across sub-samples[24]. This sensitivity analysis and other secondary analyses are available on GitHub: https://github.com/baydounm/UKB-paper21-supplementarydata. Various statistical methods are described in OSM 3 and 4. 

#### Data availability statement

The UK Biobank is an extensive biomedical database and research resource that encompasses comprehensive genetic and health data from over 500,000 individuals in the UK. The database is regularly updated and accessible to authorized research scholars globally. Requests for access to these datasets should be made at https://www.ukbiobank.ac.uk/.

## Results

**Fig. 1:**
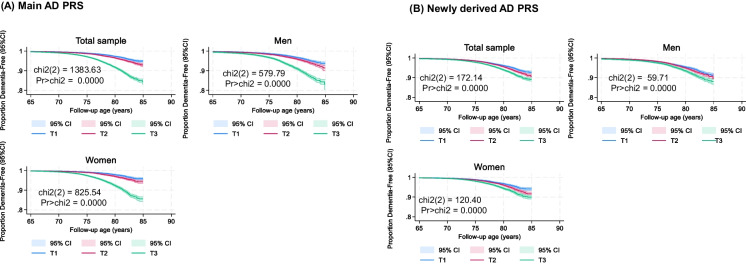
Alzheimer’s Disease polygenic risk tertiles in relation to all-cause dementia, before and after stratification by sex: UK biobank 2006–2023 (a) Main AD PRS (b) Newly derived AD PRS. *Abbreviations*: chi2 = chi-square test; CI = confidence interval; KM = Kaplan Meier; Pr = P-values; T1 = First tertile (lowest AD polygenic risk); T2 = Second teritle (medium AD polygenic risk); T3 = Uppermost tertile (Highest AD polygenic risk); UK = United Kingdom. *Note*: Chi2 refers to a log-rank test. Age of follow-up is truncated at 85y in this analysis to reduce likelihood of small sample sizes at older ages, given a baseline age range of 50-73y and up to 17 y of follow-up (between 2006–2010 and end of 2023). This restriction, which lead to a sample size of N = 202,946, was not made in the remaining parts of the analysis, that did not involve visualization with Kaplan–Meier curves. Details are provided on github: https://github.com/baydounm/UKB-paper21-supplementarydata

The study sample included 205,219 participants, with a slight majority being women (53.9%), as shown in Table 1. The mean baseline age was 60.56 years, with men slightly older than women (60.3 years). The sample was predominantly White (96.2%), with smaller proportions of Black (1.0%), South Asian (1.3%), and other racial/ethnic groups (1.5%). Socioeconomic status and household size were both higher among men, compared to women. Genetic principal components (GPCs 1–20) showed significant sex differences in some components, indicating population substructure variations across genders. While the AD PRS distributions did not vary by sex, differences by sex were detected for metabolomic principal components (*zMETAB1-15*). Dementia incidence was higher among men than woman. Results from Kaplan–Meier survival curve estimations (Fig. 1) demonstrate highly significant differences in survival distributions across the stratified AD PRS tertile groups within the study population, corroborated by highly significant log-rank tests (ch-square, 1 d.f., P < 0.001).

**Table 1:** Study sample characteristics before and after stratification by sex: UK biobank 2006–2023

	Overall (N = 205,219)	Men (N = 94,601)	Women (N = 110,618)	P_sex_
Demographic				
Baseline age, Age_base_ years	60.56 ± 0.01	60.81 ± 0.02	60.3 ± 0.02	< 0.001
Age_base_ × Age_base_	3,697.7 ± 1.4	3,728.7 ± 2.1	3,671.1 ± 2.0	< 0.001
Sex, % female	53.9%	–	–	
Race/ethnicity				
White	96.2%	96.1%	96.3%	Ref
Black	1.0%	0.8%	1.0%	< 0.001
South Asian	1.3%	1.6%	1.1%	< 0.001
Other	1.5%	1.5%	1.6%	0.046
Minority racial groups, %	3.8%	3.9%	3.7%	0.049
SES	−0.0094 ± 0.0016	+ 0.015 ± 0.002	−0.030 ± 0.002	< 0.001
Household size	2.221 ± 0.002	2.294 ± 0.003	2.159 ± 0.003	< 0.001
Genetic principal components, GPC1-20				
GPC1	−5.106 ± 0.095	−5.626 ± 0.131	−4.660 ± 0.135	< 0.001
GPC2	+ 1.318 ± 0.050	+ 1.228 ± 0.071	+ 1.396 ± 0.071	0.094
GPC3	−0.421 ± 0.027	−0.067 ± 0.041	−0.725 ± 0.037	< 0.001
GPC4	+ 0.206 ± 0.022	0.207 ± 0.033	+ 0.205 ± 0.030	0.97
GPC5	−0.280 ± 0.017	−0.322 ± 0.024	−0.244 ± 0.023	0.021
GPC6	−0.194 ± 0.009	−0.247 ± 0.011	−0.148 ± 0.014	< 0.001
GPC7	−0.103 ± 0.010	−0.030 ± 0.015	−0.166 ± 0.014	< 0.001
GPC8	−0.259 ± 0.010	−0.273 ± 0.012	−0.247 ± 0.014	0.19
GPC9	+ 0.225 ± 0.010	+ 0.226 ± 0.014	+ 0.224 ± 0.013	0.91
GPC10	+ 0.227 ± 0.008	+ 0.273 ± 0.012	+ 0.187 ± 0.010	< 0.001
GPC11	+ 0.462 ± 0.008	+ 0.492 ± 0.013	+ 0.437 ± 0.011	0.001
GPC12	−0.208 ± 0.007	−0.207 ± 0.010	−0.209 ± 0.009	0.88
GPC13	+ 0.019 ± 0.006	−0.026 ± 0.007	−0.013 ± 0.009	0.29
GPC14	+ 0.055 ± 0.007	+ 0.042 ± 0.010	+ 0.066 ± 0.010	0.10
GPC15	−0.008 ± 0.006	−0.009 ± 0.008	−0.007 ± 0.008	0.87
GPC16	+ 0.019 ± 0.007	−0.016 ± 0.010	+ 0.048 ± 0.010	< 0.001
GPC17	+ 0.004 ± 0.006	−0.012 ± 0.008	+ 0.019 ± 0.008	0.007
GPC18	−0.009 ± 0.006	−0.015 ± 0.009	−0.006 ± 0.009	0.46
GPC19	−0.001 ± 0.006	−0.007 ± 0.009	+ 0.004 ± 0.009	0.41
GPC20	+ 0.017 ± 0.006	+ 0.029 ± 0.009	+ 0.008 ± 0.008	0.096
AD PRS	1.09E-11 ± 0.002	+ 0.001 ± 0.003	−0.0014 ± 0.0030	0.50
Tertile, %				
T1	33.3	33.3	33.3	Ref
T2	33.3	33.4	33.2	0.83
T3	33.3	33.3	33.4	0.74
New AD PRS	2.52E-11 ± 0.002	+ 0.002 ± 0.003	−0.0019 ± 0.0030	0.35
Tertile, %				
T1	33.3	33.4	33.3	0.49
T2	33.3	33.2	33.4	Ref
T3	33.3	33.4	33.3	0.29
Metabolomic PCs				
zMETAB1	−3.72E-11 ± 0.002	−0.075 ± 0.003	+ 0.064 ± 0.003	< 0.001
zMETAB2	1.69E-11 ± 0.002	−0.451 ± 0.003	+ 0.386 ± 0.003	< 0.001
zMETAB3	8.15E-12 ± 0.002	+ 0.217 ± 0.003	−0.186 ± 0.003	< 0.001
zMETAB4	9.69E-11 ± 0.002	−0.399 ± 0.003	+ 0.340 ± 0.003	< 0.001
zMETAB5	−6.89E-11 ± 0.002	−0.014 ± 0.003	+ 0.012 ± 0.003	< 0.001
zMETAB6	−1.11E-11 ± 0.002	−0.273 ± 0.003	+ 0.233 ± 0.003	< 0.001
zMETAB7	−1.31E-11 ± 0.002	−0.228 ± 0.003	+ 0.195 ± 0.003	< 0.001
zMETAB8	9.50E-12 ± 0.002	+ 0.245 ± 0.003	−0.209 ± 0.003	< 0.001
zMETAB9	−2.58E-11 ± 0.002	+ 0.144 ± 0.003	−0.123 ± 0.003	< 0.001
zMETAB10	1.37E-11 ± 0.002	−0.149 ± 0.003	+ 0.128 ± 0.003	< 0.001
zMETAB11	4.22E-11 ± 0.002	−0.121 ± 0.004	+ 0.104 ± 0.003	< 0.001
zMETAB12	−9.75E-11 ± 0.002	+ 0.045 ± 0.003	−0.039 ± 0.003	< 0.001
zMETAB13	4.25E-11 ± 0.002	+ 0.041 ± 0.003	−0.035 ± 0.003	< 0.001
zMETAB14	−6.57E-11 ± 0.002	−0.057 ± 0.003	+ 0.049 ± 0.003	< 0.001
zMETAB15	−1.89E-11 ± 0.002	−0.0188 ± 0.003	+ 0.016 ± 0.003	< 0.001
Cumulative incidence, %				
All-cause dementia	2.49%	2.87%	2.18%	< 0.001
AD dementia	1.10%	1.14%	1.07%	0.10

*Abbreviations*: AD = Alzheimer’s Disease; GPC = Genetic Principal Component; PRS = Polygenic Risk Score; SE = Standard Error; UK = United Kingdom

*Notes*: No multiple imputation was carried out in this analysis. P-value is associated with the parameter for sex in bivariate linear and multinomial logistic regression analyses, with the main outcome being a continuous or categorical characteristic, respectively. (Ref) is the referent category in the multinomial logistic regression model. Values are means ± SE or percentages

Based on a series of Cox proportional hazards models (Table 2), higher AD PRS z-scores were significantly associated with increased risks of both all-cause dementia and AD dementia. Each 1-SD increase in the 2019 IGAP AD PRS was associated with a 75% higher risk of all-cause dementia (HR = 1.75, 95% CI: 1.70–1.79, p < 0.001) and a 102% higher risk of AD dementia (HR = 2.02, 95% CI: 1.95–2.09, p < 0.001). Stratified analyses indicated stronger associations in women (HRs = 1.87 for all-cause dementia and 2.12 for AD dementia) than men (HRs = 1.65 and 1.92, respectively). In the case of the newer AD PRS z-score (2022 version), associations with all-cause dementia and AD dementia were weaker in the overall sample, though sex differences were retained for all-cause dementia in the same direction as the main AD PRS.

**Table 2:** Alzheimer’s Disease polygenic risk score (AD PRS) z-score and its association with incident all-cause and AD dementia, before and after stratification by sex, Cox Proportional Hazards Models: UK biobank 2006–2023

	AD PRS z-score	
	HR with 95% CI	P_AD PRS_
2019 IGAP AD PRS		
All-cause dementia		
Overall, N = 205,219	1.75 (1.70;1.79)	< 0.001^a^
Men, N = 94,601	1.65 (1.59;1.70)	< 0.001
Women, N = 110,618	1.87 (1.80;1.93)	< 0.001
AD dementia		
Overall, N = 205,2019	2.02 (1.95; 2.09)	< 0.001^a^
Men, N = 94,601	1.92 (1.82;2.02)	< 0.001
Women, N = 110,618	2.12 (2.02; 2.22)	< 0.001
2022 AD PRS		
All-cause dementia		
Overall, N = 205,219	1.23 (1.19;1.26)	< 0.001^a^
Men, N = 94,601	1.18 (1.14;1.22)	< 0.001
Women, N = 110,618	1.28 (1.23; 1.33)	< 0.001
AD dementia		
Overall, N = 205,219	1.32 (1.26; 1.37)	< 0.001
Men, N = 94,601	1.28 (1.20; 1.35)	< 0.001
Women, N = 110,618	1.35 (1.28; 1.43)	< 0.001

*Abbreviations*: AD = Alzheimer’s Disease; GPC = Genetic Principal Component; n/a = not applicable; PRS = Polygenic Risk Score; UK = United Kingdom

^*^ All Cox proportional hazards models were adjusted for baseline age, sex, age-squared and the first 20 genetic principal components (GPC 1–20), SES and household size. Interaction between AD PRS tertiles and sex was tested, by including a 2-way interaction term in the main unstratified model. Values are hazard ratios with 95% CI. 1 SD of AD PRS is equivalent to a 1 unit increase in AD PRS in the largest available UK Biobank sample

^a^ P < 0.05 for null hypothesis that 2-way interaction between sex and AD PRS, γ = 0, in an unstratified model with main effects of sex and AD PRS included along with main effects of covariates

**Fig. 2:**
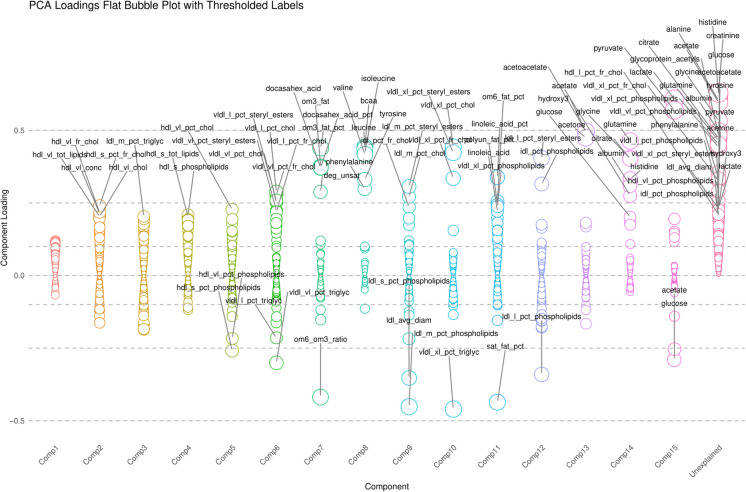
Bubble plot displaying principal components analysis of 249 plasma metabolites, with varimax rotation (k = 15 principal components). *Abbreviations*: See list of abbreviations for protein abbreviations; AD = Alzheimer’s Disease; PRS = Polygenic Risk Score

Figure 2 and supplementary datasheet 1 show the results of the principal components analysis for the 249 normalized NMR metabolites. Fifteen components were extracted and varimax rotated, with ensuing component loadings displayed (zMETAB1-15). Each component had a simple structure with metabolites loading moderately almost exclusively on them. Nevertheless, component 1 had the smallest loadings on a large number of metabolites and their ratios or percentages, explaining the largest proportion of the variance in the 249 markers combined. These components were then used for downstream analyses after normalization (z-scoring) within the final selected sample.

**Fig. 3:**
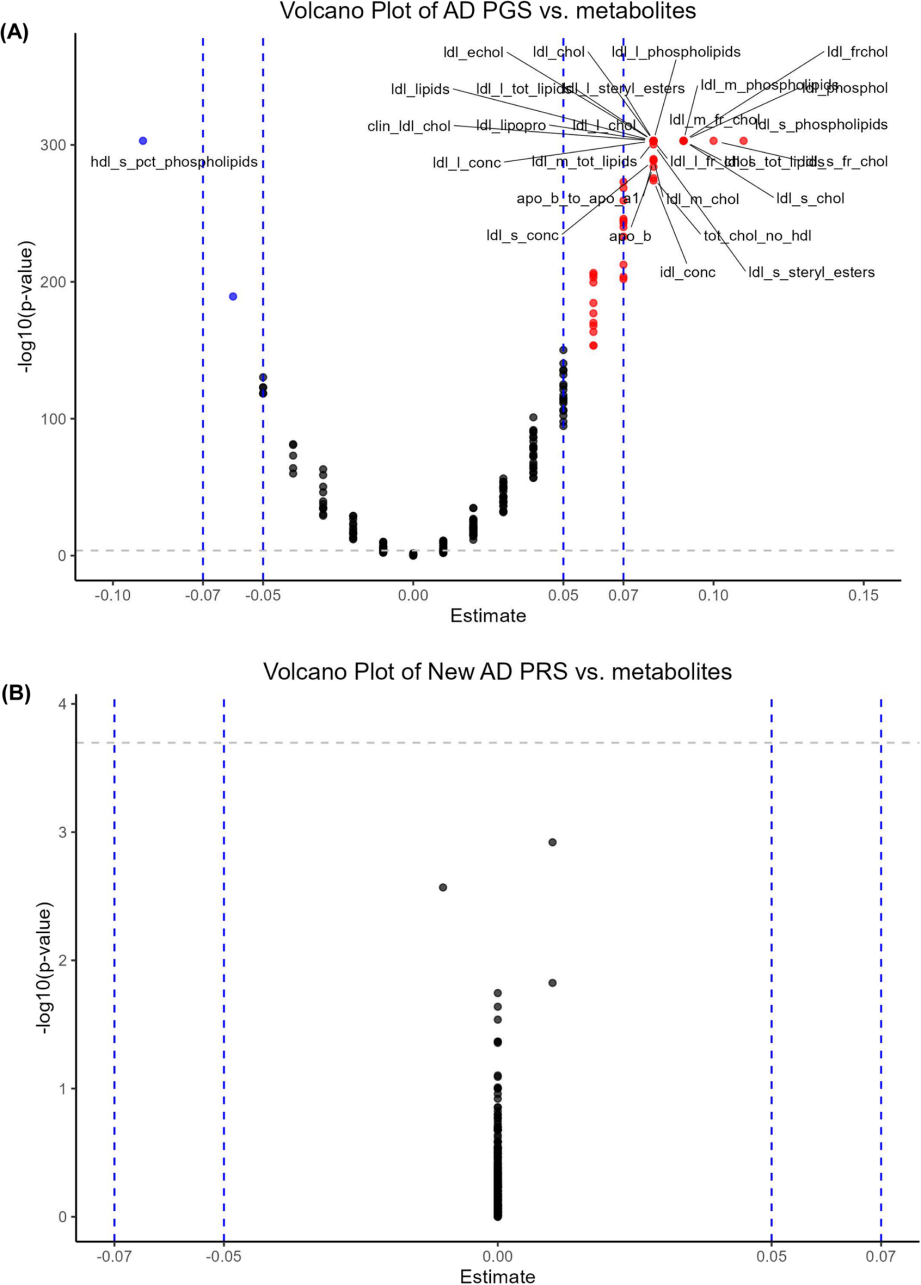
Volcano plot of plasma metabolomic biomarkers in relation to AD PRS: UK biobank 2006–2010. *Abbreviations*: See list of abbreviations for protein abbreviations; AD = Alzheimer’s Disease; PRS = Polygenic Risk Score. *Note*: Based on a series of multiple linear regression models, with main predictor being AD PRS and the outcome being each of 249 plasma metabolomic biomarkers (square-root transformed, z-scored). The y-axis is the predictor’s associated p-value on a -Log10 scale and the X-axis is the β coefficient (effect of AD PRS exposure on standardized z-scores of plasma metabolomic markers) from the multiple linear regression models. An estimate with a Bonferroni corrected p-value < 0.05 are marked by a different color and the plasma proteomic marker abbreviation is added for relatively stronger effect size of > 0.050 in absolute value (See UKB showcase URL: https://biobank.ndph.ox.ac.uk/showcase/). Details are provided on github: https://github.com/baydounm/UKB-paper21-supplementarydata

Figure 3 displays two volcano plots each with 249 multivariable-adjusted linear regression with main predictor being AD PRS z-score and the main outcome being each of 249 metabolites (and ratios of metabolites). Using the 2019 IGAP AD PRS metric, the strongest associations were between AD genetic risk and several LDL-related metabolites in addition to ApoB, ApoB to ApoA1 ratio, and IDL concentration (See supplementary datasheet 2). The strongest effect on the positive side was ldl_s_phospholipids (Phospholipids in Small LDL), with an effect size of + 0.11. On the negative effect size of the plot, the strongest inverse relationship between AD PRS and the metabolome was detected for hdl_s_pct_phospholipids (Phospholipids to Total Lipids in Small HDL percentage) with an effect size of −0.09. This indicates that genetic risk for AD is modestly associated with increased phosphorylation of small LDL particles, possibly coupled with reduced phosphorylation of small HDL particles. None of the relationships between the new AD PRS and the metabolome passed Bonferroni correction (See supplementary datasheet 2). As a result, no post hoc mediational analysis will be performed for the new version of AD PRS.

Using the main version of AD PRS (IGAP-2019), the total effect of AD genetic risk on dementia risk was partially but modestly mediated through specific metabolomic principal components, based on a series of generalized structural equations models with post-estimated mediated effects (Table 3). Significant findings included zMETAB1 contributing to reduced dementia risk, though being positively related to AD PRS (IGAP-2019). This resulted in a protective mediation whereby the direct effect was stronger than the total effect by ~ 1.5% when zMETAB1 was increased from its mean to a mean + 1SD. The same was observed for zMETAB5, whereby the mediated percentage was −1.63%. Nevertheless, for this analysis with principal components, most mediation effects were small, suggesting that the majority of AD PRS impact on dementia risk operates independently of the tested metabolomic components. The results were also weak in terms of mediation when examining AD as an outcome instead of all-cause dementia (Table 4).

**Table 3:** Generalized structural equations model for the effect of AD PGS (z-scored) on all-cause dementia risk through plasma metabolomic principal components (z-scored) (k = 15; N_max_ = 205,219): UK biobank 2006–2023

	Beta	SE	Z	P	LCL	UCL	METABOLOMIC Principal component
zAD_PGS$\rightarrow$ Dementia	** + 0.567**	**0.012**	**46.67**	** < 0.001**	**0.542**	**0.591**	zMETAB1
zAD_PGS$\rightarrow$ zMETAB	** + 0.058**	**0.002**	**26.57**	** < 0.001**	**0.054**	**0.062**	zMETAB1
zMETAB$\rightarrow$ Dementia	**−0.135**	**0.014**	**−9.54**	** < 0.001**	**−0.163**	**−0.107**	zMETAB1
Indirect	**−0.0078**	**0.0009**	**−8.98**	** < 0.001**	**−0.0096**	**−0.0061**	zMETAB1
Total effect	** + 0.559**	**0.0121**	**46.09**	** < 0.001**	**0.5351**	**0.5826**	zMETAB1
% mediated	**−1.404**	**0.160**	**−8.79**	** < 0.001**	**−1.717**	**−1.091**	zMETAB1
zAD_PGS$\rightarrow$ Dementia	** + 0.560**	**0.012**	**46.17**	** < 0.001**	**0.536**	**0.585**	zMETAB2
zAD_PGS$\rightarrow$ zMETAB	** + 0.027**	**0.020**	**13.64**	** < 0.001**	**0.023**	**0.031**	zMETAB2
zMETAB$\rightarrow$ Dementia	−0.004	0.0156	−0.26	0.79	−0.0347	0.0265	zMETAB2
Indirect	−0.0001	0.0004	−0.26	0.79	−0.0009	0.0007	zMETAB2
Total effect	** + 0.560**	**0.0121**	**46.20**	** < 0.001**	** + 0.536**	** + 0.584**	zMETAB2
% mediated	−0.020	0.076	−0.26	0.79	−0.167	0.128	zMETAB2
zAD_PGS$\rightarrow$ Dementia	** + 0.562**	**0.012**	**46.34**	** < 0.001**	** + 0.538**	**0.586**	zMETAB3
zAD_PGS$\rightarrow$ zMETAB	**−0.032**	**0.002**	**−15.1**	** < 0.001**	**−0.036**	**−0.028**	zMETAB3
zMETAB$\rightarrow$ Dementia	** + 0.071**	**0.014**	**5.02**	** < 0.001**	** + 0.043**	**0.098**	zMETAB3
Indirect	**−0.0023**	**0.0005**	**−4.76**	** < 0.001**	**−0.003**	**−0.001**	zMETAB3
Total effect	** + 0.560**	**0.012**	**46.18**	** < 0.001**	** + 0.536**	** + 0.584**	zMETAB3
% mediated	**−0.407**	**0.086**	**−4.73**	** < 0.001**	**−0.575**	**−0.238**	zMETAB3
zAD_PGS$\rightarrow$ Dementia	** + 0.559**	**0.012**	**46.12**	** < 0.001**	** + 0.535**	** + 0.583**	zMETAB4
zAD_PGS$\rightarrow$ zMETAB	***−0.003***	***0.002***	***−1.76***	***0.079***	***−0.0076***	***0.0004***	zMETAB4
zMETAB$\rightarrow$ Dementia	**−0.1008**	**0.0153**	**−6.58**	** < 0.001**	**−0.131**	**−0.071**	zMETAB4
Indirect	** + *0.0004***	***0.0002***	***1.70***	***0.090***	***−0.0001***	** + *0.0008***	zMETAB4
Total effect	** + 0.559**	**0.012**	**46.15**	** < 0.001**	** + 0.535**	**0.583**	zMETAB4
% mediated	** + *0.065***	***0.038***	***1.70***	***0.090***	***−0.010***	***0.140***	zMETAB4
zAD_PGS$\rightarrow$ Dementia	** + 0.569**	**0.012**	**46.73**	** < 0.001**	** + 0.545**	**0.592**	zMETAB5
zAD_PGS$\rightarrow$ zMETAB	** + 0.082**	**0.002**	**37.73**	** < 0.001**	** + 0.078**	**0.086**	zMETAB5
zMETAB$\rightarrow$ Dementia	**−0.110**	**0.014**	**−7.95**	** < 0.001**	**−0.138**	**−0.083**	zMETAB5
Indirect	**−0.009**	**0.001**	**−7.78**	** < 0.001**	**−0.011**	**−0.007**	zMETAB5
Total effect	** + 0.559**	**0.012**	**46.15**	** < 0.001**	**0.535**	**0.583**	zMETAB5
% mediated	**−1.63**	**0.21**	**−7.66**	** < 0.001**	**−2.05**	**−1.21**	zMETAB5
zAD_PGS$\rightarrow$ Dementia	** + 0.560**	**0.012**	**46.16**	** < 0.001**	** + 0.537**	** + 0.584**	zMETAB6
zAD_PGS$\rightarrow$ zMETAB	** + 0.032**	**0.002**	**14.96**	** < 0.001**	** + 0.028**	** + 0.036**	zMETAB6
zMETAB$\rightarrow$ Dementia	−0.011	0.014	−0.75	0.45	−0.039	+ 0.017	zMETAB6
Indirect	−0.0003	0.0005	−0.75	0.45	−0.00012	+ 0.0005	zMETAB6
Total effect	**0.560**	** + 0.012**	**46.20**	** < 0.001**	**0.536**	**0.584**	**zMETAB6**
% mediated	−0.061	0.08	−0.75	0.45	−0.222	0.099	zMETAB6
zAD_PGS$\rightarrow$ Dementia	** + 0.560**	**0.012**	**46.21**	** < 0.001**	**0.536**	**0.583**	zMETAB7
zAD_PGS$\rightarrow$ zMETAB	** + 0.007**	**0.0021**	**3.49**	** < 0.001**	**0.003**	**0.011**	zMETAB7
zMETAB$\rightarrow$ Dementia	**−0.076**	**0.015**	**−5.17**	** < 0.001**	**−0.105**	**−0.047**	zMETAB7
Indirect	**−0.00056**	**0.00019**	**−2.89**	**0.004**	**−0.0009**	**−0.0002**	zMETAB7
Total effect	** + 0.560**	**0.0121**	**46.12**	** < 0.001**	** + 0.536**	**0.583**	zMETAB7
% mediated	**−0.099**	**0.035**	**−2.88**	**0.004**	**−0.167**	**−0.032**	zMETAB7
zAD_PGS$\rightarrow$ Dementia	**−0.559**	**0.012**	**46.12**	** < 0.001**	**0.535**	**0.582**	zMETAB8
zAD_PGS$\rightarrow$ zMETAB	**−0.009**	**0.002**	**−4.00**	** < 0.001**	**−0.013**	**−0.004**	zMETAB8
zMETAB$\rightarrow$ Dementia	**−0.065**	**0.015**	**−4.41**	** < 0.001**	**−0.094**	**−0.036**	zMETAB8
Indirect	** + 0.0006**	**0.0002**	**2.96**	**0.003**	** + 0.0002**	** + 0.0009**	zMETAB8
Total effect	** + 0.560**	**0.0121**	**46.2**	** < 0.001**	** + 0.536**	** + 0.583**	zMETAB8
% mediated	** + 0.099**	**0.034**	**2.96**	**0.003**	** + 0.0336**	**0.1660**	zMETAB8
zAD_PGS$\rightarrow$ Dementia	** + 0.557**	**0.012**	**45.92**	** < 0.001**	** + 0.534**	** + 0.581**	zMETAB9
zAD_PGS$\rightarrow$ zMETAB	**−0.035**	**0.002**	**−16.05**	** < 0.001**	**−0.039**	**−0.031**	zMETAB9
zMETAB$\rightarrow$ Dementia	**−0.076**	**0.015**	**−5.25**	** < 0.001**	**−0.105**	**−0.048**	zMETAB9
Indirect	** + 0.0027**	** + 0.0005**	**4.99**	** < 0.001**	** + 0.0016**	** + 0.0037**	zMETAB9
Total effect	** + 0.5600**	**0.0121**	**46.17**	** < 0.001**	** + 0.536**	** + 0.584**	zMETAB9
% mediated	** + 0.477**	**0.096**	**4.96**	** < 0.001**	** + 0.289**	** + 0.666**	zMETAB9
zAD_PGS$\rightarrow$ Dementia	** + 0.560**	**0.012**	**46.21**	** < 0.001**	** + 0.536**	** + 0.584**	zMETAB10
zAD_PGS$\rightarrow$ zMETAB	+ 0.0005	0.002	0.21	0.83	−0.003	0.005	zMETAB10
zMETAB$\rightarrow$ Dementia	**−0.031**	**0.014**	**−2.19**	**0.029**	**−0.059**	**−0.003**	zMETAB10
Indirect	−0.00001	0.00006	−0.21	0.83	−0.0001	0.0001	zMETAB10
Total effect	** + 0.560**	**0.012**	**46.20**	** < 0.001**	** + 0.536**	** + 0.584**	zMETAB10
% mediated	−0.003	0.012	−0.21	0.83	−0.027	+ 0.021	zMETAB10
zAD_PGS$\rightarrow$ Dementia	** + 0.562**	**0.012**	**46.32**	** < 0.001**	** + 0.538**	** + 0.586**	zMETAB11
zAD_PGS$\rightarrow$ zMETAB	**−0.013**	**0.002**	**6.17**	** < 0.001**	** + 0.009**	** + 0.018**	zMETAB11
zMETAB$\rightarrow$ Dementia	**−0.096**	**0.014**	**−7.07**	** < 0.001**	**−0.122**	**−0.069**	zMETAB11
Indirect	**−0.0013**	**0.0003**	**−4.65**	** < 0.001**	**−0.0018**	**−0.0007**	zMETAB11
Total effect	** + 0.561**	**0.012**	**46.21**	** < 0.001**	**0.537**	**0.584**	zMETAB11
% mediated	**−0.229**	**0.049**	**−4.62**	** < 0.001**	**−0.326**	**−0.131**	zMETAB11
zAD_PGS$\rightarrow$ Dementia	** + 0.561**	**0.012**	**46.24**	** < 0.001**	** + 0.537**	** + 0.584**	zMETAB12
zAD_PGS$\rightarrow$ zMETAB	** + 0.009**	**0.002**	**4.00**	** < 0.001**	** + 0.004**	** + 0.013**	zMETAB12
zMETAB$\rightarrow$ Dementia	**−0.048**	**0.014**	**−3.45**	**0.001**	**−0.075**	**−0.020**	zMETAB12
Indirect	**−0.0004**	**0.0001**	**−2.61**	**0.009**	**−0.0007**	**−0.0001**	zMETAB12
Total effect	** + 0.560**	**0.012**	**46.21**	** < 0.001**	** + 0.537**	** + 0.584**	zMETAB12
% mediated	**−0.076**	**0.029**	**−2.61**	**0.009**	**−0.133**	**−0.019**	zMETAB12
zAD_PGS$\rightarrow$ Dementia	** + 0.560**	**0.012**	**46.14**	** < 0.001**	** + 0.536**	** + 0.583**	zMETAB13
zAD_PGS$\rightarrow$ zMETAB	** + 0.0125**	**0.0022**	**5.71**	** < 0.001**	** + 0.008**	** + 0.017**	zMETAB13
zMETAB$\rightarrow$ Dementia	** + 0.053**	**0.014**	**3.83**	** < 0.001**	** + 0.026**	** + 0.080**	zMETAB13
Indirect	** + 0.00066**	**0.0002**	**3.18**	**0.001**	** + 0.0002**	** + 0.0011**	zMETAB13
Total effect	** + 0.560**	**0.012**	**46.20**	** < 0.001**	** + 0.536**	** + 0.584**	zMETAB13
% mediated	** + 0.119**	**0.038**	**3.18**	**0.001**	** + 0.046**	** + 0.193**	zMETAB13
zAD_PGS$\rightarrow$ Dementia	** + 0.560**	**0.012**	**46.20**	** < 0.001**	** + 0.536**	** + 0.584**	zMETAB14
zAD_PGS$\rightarrow$ zMETAB	−0.0026	0.0022	−1.17	0.24	−0.0069	+ 0.0017	zMETAB14
zMETAB$\rightarrow$ Dementia	+ 0.0023	0.0140	0.16	0.87	−0.0252	+ 0.0297	zMETAB14
Indirect	+ 0.00000	0.00004	−0.16	0.87	−0.00008	0.00007	zMETAB14
Total effect	** + 0.560**	**0.012**	**46.20**	** < 0.001**	** + 0.536**	** + 0.585**	zMETAB14
% mediated	−0.0010	0.0065	−0.16	0.87	−0.014	+ 0.0117	zMETAB14
zAD_PGS$\rightarrow$ Dementia	** + 0.560**	**0.0121**	**46.18**	** < 0.001**	**0.536**	**0.584**	zMETAB15
zAD_PGS$\rightarrow$ zMETAB	**−0.0068**	**0.0022**	**−3.08**	**0.002**	**−0.011**	**−0.003**	zMETAB15
zMETAB$\rightarrow$ Dementia	−0.0160	0.0146	−1.10	0.27	−0.429	0.003	zMETAB15
Indirect	+ 0.0001	0.0001	1.04	0.30	−0.00010	0.00031	zMETAB15
Total effect	** + 0.560**	**0.012**	**46.12**	** < 0.001**	** + 0.536**	** + 0.583**	zMETAB15
% mediated	+ 0.019	0.019	2.04	0.30	−0.017	+ 0.056	zMETAB15

*Abbreviations*: AD = Alzheimer’s Disease; ereri_cde = excess relative risk due to neither mediation nor interaction or controlled direct effect; PRS = Polygenic Risk Score; UK = United Kingdom. The total effect is interpreted as a Ln(hazard ratio). AD PRS was re-normalized within the selected sample as a Z-score, and therefore a unit increase is equivalent to 1 SD increase in AD polygenic risk. Models were adjusted for age, age-squared, sex and the first 20 genetic principal components (GPC 1–20), SES and household size. Mediation analyses were based on generalized structural equation modeling with Weibull distribution. % mediated = proportion of total effect explained by indirect pathway through metabolomic component. Negative % mediated values indicate protective mediation (i.e., indirect effect opposes total effect)

**Table 4:** Generalized structural equations model for the effect of AD PGS (z-scored) on AD dementia risk through plasma metabolomic principal components (z-scored) (k = 15; N_max_ = 205,219): UK biobank 2006–2023

	Beta	SE	Z	P	LCL	UCL	METABOLOMIC Principal component
zAD_PGS$\rightarrow$ AD	** + 0.710**	**0.018**	**40.15**	**0.000**	** + 0.675**	** + 0.745**	zMETAB1
zAD_PGS$\rightarrow$ zMETAB	** + 0.058**	**0.002**	**26.59**	**0.000**	** + 0.054**	** + 0.063**	zMETAB1
zMETAB$\rightarrow$ AD	**−0.098**	**0.021**	**−4.65**	**0.000**	**−0.140**	**−0.057**	zMETAB1
Indirect	**−0.006**	**0.001**	**−4.58**	**0.000**	**−0.008**	**−0.003**	zMETAB1
Total effect	** + 0.704**	**0.018**	**39.88**	**0.000**	** + 0.670**	** + 0.739**	zMETAB1
% mediated	**−0.813**	**0.179**	**−4.54**	**0.000**	**−1.164**	**−0.462**	zMETAB1
zAD_PGS$\rightarrow$ AD	** + 0.704**	**0.018**	**39.83**	**0.000**	** + 0.669**	** + 0.739**	zMETAB2
zAD_PGS$\rightarrow$ zMETAB	** + 0.027**	**0.002**	**13.68**	**0.000**	** + 0.023**	** + 0.031**	zMETAB2
zMETAB$\rightarrow$ AD	** + *0.040***	***0.023***	***1.71***	***0.086***	***−0.006***	** + *0.086***	zMETAB2
Indirect	** + *0.001***	***0.0006***	***1.70***	***0.089***	***−0.0002***	***0.0023***	zMETAB2
Total effect	** + 0.705**	**0.018**	**39.93**	**0.000**	** + 0.671**	** + 0.740**	zMETAB2
% mediated	** + *0.153***	***0.090***	***1.70***	***0.089***	***−0.024***	** + *0.331***	zMETAB2
zAD_PGS$\rightarrow$ AD	** + 0.706**	**0.018**	**39.93**	**0.000**	** + 0.671**	** + 0.740**	zMETAB3
zAD_PGS$\rightarrow$ zMETAB	**−0.032**	**0.0021**	**−15.13**	**0.000**	**−0.036**	**−0.028**	zMETAB3
zMETAB$\rightarrow$ AD	+ 0.015	0.022	0.69	0.489	−0.027	+ 0.057	zMETAB3
Indirect	−0.0005	0.0007	−0.69	0.490	−0.0019	0.0009	zMETAB3
Total effect	** + 0.705**	**0.0177**	**39.93**	**0.000**	**0.671**	**0.740**	zMETAB3
% mediated	−0.069	0.099	−0.69	0.490	−0.263	0.126	zMETAB3
zAD_PGS$\rightarrow$ AD	** + 0.705**	**0.018**	**39.91**	**0.000**	**0.670**	**0.740**	zMETAB4
zAD_PGS$\rightarrow$ zMETAB	***−0.004***	***0.002***	***−1.75***	***0.080***	***−0.008***	** + *0.000***	zMETAB4
zMETAB$\rightarrow$ AD	−0.036	0.023	−1.59	0.11	−0.082	0.009	zMETAB4
Indirect	+ 0.0001	0.0001	1.18	0.24	−0.00008	0.0004	zMETAB4
Total effect	**0.705**	**0.017**	**39.91**	**0.000**	**0.671**	**0.740**	zMETAB4
% mediated	0.019	0.016	1.18	0.24	−0.012	0.050	zMETAB4
zAD_PGS$\rightarrow$ AD	** + 0.709**	**0.018**	**39.96**	**0.000**	** + 0.674**	** + 0.743**	zMETAB5
zAD_PGS$\rightarrow$ zMETAB	** + 0.082**	**0.002**	**37.69**	**0.000**	** + 0.078**	** + 0.087**	zMETAB5
zMETAB$\rightarrow$ AD	**−0.045**	**0.021**	**−2.11**	**0.035**	**−0.087**	**−0.003**	zMETAB5
Indirect	**−0.004**	**0.002**	**−2.10**	**0.036**	**−0.007**	**−0.000**	zMETAB5
Total effect	** + 0.705**	**0.018**	**39.92**	**0.000**	** + 0.670**	** + 0.740**	zMETAB5
% mediated	**−0.525**	**0.250**	**−2.10**	**0.036**	**−1.015**	**−0.035**	zMETAB5
zAD_PGS$\rightarrow$ AD	**+ 0.704**	**0.018**	**39.84**	**0.000**	** + 0.670**	**+ 0.739**	zMETAB6
zAD_PGS$\rightarrow$ zMETAB	** + 0.032**	**0.002**	**14.99**	**0.000**	** + 0.028**	** + 0.036**	zMETAB6
zMETAB$\rightarrow$ AD	+ 0.035	0.022	1.62	0.106	−0.007	+ 0.078	zMETAB6
Indirect	+ 0.001	0.001	1.61	0.108	**−0.000**	** + 0.002**	zMETAB6
Total effect	**+ 0.705**	**0.018**	**39.93**	**0.000**	**+ 0.671**	** + 0.740**	zMETAB6
% mediated	**+ 0.159**	**0.099**	**1.61**	**0.108**	**−0.035**	** + 0.352**	zMETAB6
zAD_PGS$\rightarrow$ AD	**+ 0.705**	**0.018**	**39.93**	**0.000**	** + 0.671**	** + 0.740**	zMETAB7
zAD_PGS$\rightarrow$ zMETAB	**+ 0.007**	**0.002**	**3.50**	**0.000**	**+ 0.003**	** + 0.011**	zMETAB7
zMETAB$\rightarrow$ AD	−0.044	0.022	−2.00	0.046	−0.087	−0.001	zMETAB7
Indirect	−0.000	0.000	−1.73	0.083	−0.001	0.000	zMETAB7
Total effect	** + 0.705**	**0.018**	**39.91**	**0.000**	**+ 0.670**	**0.740**	zMETAB7
% mediated	**−0.046**	**0.027**	−1.73	0.083	−0.098	+ 0.006	zMETAB7
zAD_PGS$\rightarrow$ AD	**+ 0.704**	**0.018**	**39.85**	**0.000**	** + 0.669**	** + 0.738**	zMETAB8
zAD_PGS$\rightarrow$ zMETAB	**−0.009**	**0.002**	**−4.00**	**0.000**	**−0.013**	**−0.004**	zMETAB8
zMETAB$\rightarrow$ AD	**−0.092**	**0.022**	**−4.16**	**0.000**	**−0.136**	**−0.049**	zMETAB8
Indirect	** + 0.001**	**0.000**	**2.88**	**0.004**	** + 0.000**	** + 0.001**	zMETAB8
Total effect	** + 0.704**	**0.018**	**39.90**	**0.000**	** + 0.670**	** + 0.739**	zMETAB8
% mediated	** + 0.113**	**0.039**	**2.87**	**0.004**	** + 0.036**	** + 0.190**	zMETAB8
zAD_PGS$\rightarrow$ AD	** + 0.703**	**0.018**	**39.75**	**0.000**	** + 0.668**	** + 0.738**	zMETAB9
zAD_PGS$\rightarrow$ zMETAB	**−0.035**	**0.002**	**−16.05**	**0.000**	**−0.039**	**−0.031**	zMETAB9
zMETAB$\rightarrow$ AD	**−0.065**	**0.022**	**−2.97**	**0.003**	**−0.108**	**−0.108**	zMETAB9
Indirect	** + 0.002**	** + 0.001**	**2.92**	**0.004**	** + 0.001**	** + 0.004**	zMETAB9
Total effect	** + 0.705**	** + 0.018**	**39.91**	**0.000**	** + 0.671**	** + 0.740**	zMETAB9
% mediated	** + 0.324**	** + 0.111**	**2.91**	**0.004**	** + 0.106**	** + 0.542**	zMETAB9
zAD_PGS$\rightarrow$ AD	** + 0.705**	** + 0.018**	**39.93**	**0.000**	** + 0.671**	** + 0.740**	zMETAB10
zAD_PGS$\rightarrow$ zMETAB	+ 0.001	+ 0.002	0.24	0.808	−0.004	+ 0.005	zMETAB10
zMETAB$\rightarrow$ AD	+ 0.008	+ 0.021	0.35	0.723	−0.034	+ 0.049	zMETAB10
Indirect	+ 0.000	+ 0.000	0.20	0.841	−0.000	+ 0.000	zMETAB10
Total effect	** + 0.705**	** + 0.018**	**39.93**	**0.000**	** + 0.671**	** + 0.740**	zMETAB10
% mediated	+ 0.001	+ 0.003	0.20	0.841	−0.005	+ 0.006	zMETAB10
zAD_PGS$\rightarrow$ AD	** + 0.707**	** + 0.018**	**39.97**	**0.000**	** + 0.672**	** + 0.741**	zMETAB11
zAD_PGS$\rightarrow$ zMETAB	** + 0.013**	** + 0.002**	**6.13**	**0.000**	** + 0.009**	** + 0.018**	zMETAB11
zMETAB$\rightarrow$ AD	**−0.057**	** + 0.021**	**−2.74**	**0.006**	**−0.098**	**−0.016**	zMETAB11
Indirect	**−0.001**	** + 0.000**	**−2.50**	**0.012**	**−0.001**	**−0.000**	zMETAB11
Total effect	** + 0.706**	** + 0.018**	**39.94**	**0.000**	** + 0.671**	** + 0.740**	zMETAB11
% mediated	**−0.108**	** + 0.043**	**−2.50**	**0.012**	** + 0.193**	**−0.023**	zMETAB11
zAD_PGS$\rightarrow$ AD	** + 0.706**	** + 0.018**	**39.97**	**0.000**	** + 0.671**	** + 0.741**	zMETAB12
zAD_PGS$\rightarrow$ zMETAB	** + 0.008**	** + 0.002**	**3.75**	**0.000**	** + 0.004**	** + 0.013**	zMETAB12
zMETAB$\rightarrow$ AD	**−0.074**	** + 0.021**	**−3.52**	**0.000**	**−0.116**	**−0.033**	zMETAB12
Indirect	**−0.001**	** + 0.000**	**−2.57**	**0.010**	**−0.001**	**−0.000**	zMETAB12
Total effect	** + 0.705**	** + 0.018**	**39.94**	**0.000**	** + 0.671**	** + 0.740**	zMETAB12
% mediated	**−0.087**	**0.034**	**−2.56**	**0.010**	**−0.154**	**−0.020**	zMETAB12
zAD_PGS$\rightarrow$ AD	** + 0.705**	** + 0.018**	**39.91**	**0.000**	** + 0.670**	** + 0.740**	zMETAB13
zAD_PGS$\rightarrow$ zMETAB	** + 0.013**	** + 0.002**	**5.70**	**0.000**	** + 0.008**	** + 0.017**	zMETAB13
zMETAB$\rightarrow$ AD	+ 0.033	+ 0.021	1.57	0.116	−0.008	+ 0.074	zMETAB13
Indirect	+ 0.000	+ 0.000	1.52	0.130	−0.000	+ 0.001	zMETAB13
Total effect	** + 0.705**	** + 0.018**	**39.93**	**0.000**	**+ 0.671**	** + 0.740**	zMETAB13
% mediated	+ 0.059	+ 0.039	1.51	0.130	−0.017	+ 0.134	zMETAB13
zAD_PGS$\rightarrow$ AD	** + 0.705**	** + 0.018**	**39.93**	**0.000**	** + 0.671**	** + 0.740**	zMETAB14
zAD_PGS$\rightarrow$ zMETAB	−0.003	0.002	−1.17	0.244	−0.007	0.002	zMETAB14
zMETAB$\rightarrow$ AD	+ 0.026	+ 0.021	1.24	0.213	−0.015	+ 0.068	zMETAB14
Indirect	−0.000	+ 0.000	−0.85	0.395	−0.000	+ 0.000	zMETAB14
Total effect	** + 0.705**	** + 0.018**	**39.92**	**0.000**	** + 0.671**	** + 0.740**	zMETAB14
% mediated	−0.010	+ 0.011	−0.85	0.395	−0.032	+ 0.013	zMETAB14
zAD_PGS$\rightarrow$ AD	** + 0.705**	** + 0.018**	**39.92**	**0.000**	** + 0.671**	** + 0.740**	zMETAB15
zAD_PGS$\rightarrow$ zMETAB	**−0.007**	**+ 0.002**	**−3.10**	**0.002**	**−0.011**	**−0.003**	zMETAB15
zMETAB$\rightarrow$ AD	−0.021	+ 0.022	−0.94	0.346	−0.064	+ 0.022	zMETAB15
Indirect	+ 0.000	+ 0.000	0.90	0.367	−0.000	+ 0.000	zMETAB15
Total effect	** + 0.705**	** + 0.018**	**39.93**	**0.000**	** + 0.671**	** + 0.740**	zMETAB15
% mediated	+ 0.020	+ 0.022	0.90	0.367	−0.023	+ 0.063	zMETAB15

*Abbreviations*: AD = Alzheimer’s Disease; ereri_cde = excess relative risk due to neither mediation nor interaction or controlled direct effect; PRS = Polygenic Risk Score; UK = United Kingdom. The total effect is interpreted as a Ln(hazard ratio). AD PRS was re-normalized within the selected sample as a Z-score, and therefore a unit increase is equivalent to 1 SD increase in AD polygenic risk. Models were adjusted for age, age-squared, sex and the first 20 genetic principal components (GPC 1–20), SES and household size. Mediation analyses were based on generalized structural equation modeling with Weibull distribution. % mediated = proportion of total effect explained by indirect pathway through metabolomic component. Negative % mediated values indicate protective mediation (i.e., indirect effect opposes total effect)

Using four-way decomposition models on 10 equally sized random samples, the results of GSEM were corroborated with most of the effect that was mediated being a pure indirect effect specifically through zMETAB1 and zMETAB5. A meta-analysis across the 10 chunks of data yielded a statistically significant PIE at type I error of 0.05 that is comparable to the indirect effect obtained in the GSEM models, with very little heterogeneity across the 10 sub-samples, implying consistency upon replication (Figure S2 and supplementary datasheet 3).

**Fig. 4:**
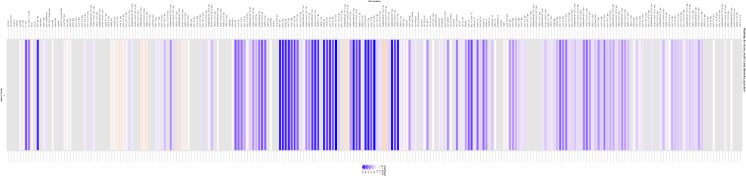
Heatmap plots of percentage mediated from a series of 249 generalized structural equations models examining mediated effects of each metabolites for the total effect of the main AD PRS on all-cause dementia. *Abbreviations*: AD = Alzheimer’s Disease; PRS = Polygenic Risk Score. *Notes*: These are based on generalized structural equations models with each metabolite entered as an alternative mediator while adjusting for potential confounding exogenous variables. Red colored lines indicate a positive or consistent mediation whereby the indirect effect and the total effect have the same direction of association; Blue colored lines indicate a negative or inconsitent mediation whereby the indirect effect and the total effect have diverging directions of association (i.e. one is positive and the other is negative). Gray colored lines indicate non-significant mediating effect with p > 0.05. Details provided in supplementary datasheet 4 (nlcom_model 1: portion mediated; 2: total effect; 3: percentage mediated)

A list of metabolomic measures that displayed a 2% or higher percent mediated in absolute value was created using results shown in Fig. 4 and detailed in supplementary datasheet 4. While for the AD outcome and the IGAP-2019 AD PRS, none of the metabolites fulfilled this condition, 24 metabolites were deemed significant mediators for the dementia outcome, specifically displaying protective mediation. Such mediation occurs when an exposure (AD PRS) is positively associated with the outcome, also increase the level of a mediator which in turn is inversely related to the final outcome, all-cause dementia. The nature of these metabolites related to lipids and lipoprotein particles, specifically Low-Density Lipoproteins (LDL) and their subclasses (large, medium, and small LDL particles). Key commonalities include LDL-centric measures related to total cholesterol, phospholipids, cholesteryl esters, and free cholesterol, subclasses of LDL focusing on different particle sizes and quantifying their specific lipid content and concentration, and lipoprotein metabolism focusing on metabolic and clinical biomarkers often used to assess cardiovascular risk.

## Discussion

### Summary of findings

This UK Biobank-based study found that higher AD risk is associated with increased risks of all-cause dementia and AD dementia, with more pronounced effects in women. Metabolomic analyses identified lipid-related markers as key mediators, revealing positive associations between LDL phospholipids and AD genetic risk, and inverse associations with HDL phospholipids.. Mediation analyses showed that LDL-related metabolomic components partially mediated dementia risk, although most genetic risk operated independently of metabolites. The study emphasizes sex-specific associations and the mediating role of metabolites between AD PRS and dementia risk.

### AD PRS, APOE4 status and dementia

Although *APOE* ε4 is a strong genetic risk factor for the development of AD, other genetic variants with small effect sizes associated with AD can be aggregated into a PRS and used for prediction. AD PRSs have been shown to predict dementia and AD [25, 26] as well as, amyloid-beta deposition and cognitive function in at-risk populations [27–29]. Consistent with these findings, we found that AD PRS was associated with the risk of both all-cause and AD dementia. Therefore, our data in a large population based study builds upon previous studies examining the relationship between the association of AD PRS and dementia.

### Metabolomics and AD PRS

Biomarker-based and precision medicine approaches are being explored to advance precision therapy for AD [10, 30]. The integration of omics approaches, such as metabolomics and transcriptomics, with genome-wide association studies (GWAS) has been highlighted[31]. Blood-based omics and metabolic signatures have shown that AD genetic risk manifests in multi-omic blood profiles in healthy individuals aged 18-90y [32]. Dysregulated plasma lipidomes have been found in AD, with strong associations between lipid profiles and disease risk genes [33]. The causal relationships between blood metabolites, cognitive factors, and AD have been explored, with genetic overlap between AD and blood lipid levels suggesting shared biological pathways [34]. Findings from the current study support previous results suggesting that HDLs have a protective effect on AD risk, whereas LDLs confer additional risk independent of APOE [35–37]. Although the positive and inverse associations between LDLs and HDLs, respectively, suggests that the genetics underlying AD may increase augment lipids, it remains plausible that these lipid levels may be altered in those with preclinical and clinical AD as a result of the evolving disease process. The mediation analyses conducted as part of the current study highlights these lipids as potential mediators linking AD genetic risk to dementia risk.

### Metabolomics vs. dementia incidence

Omics-based biomarker discovery for AD has been a topic of interest in recent years. Researchers have used various omics technologies, including genomics, transcriptomics, proteomics, and metabolomics, to discover biomarkers for AD [31]. These biomarkers have shown potential for early diagnosis and therapeutic targets [31]. For example, metabolomics has been linked to dementia risk and identified pathways involved in disease progression. Studies have also found specific blood metabolites that predict mild cognitive impairment in the Latino population [38]. Metabolomics has also been used to study microglia, providing insights into their role in AD pathogenesis, therapeutic strategies, and biomarker development [39]. The emerging field of nutritional lipidomics has also been explored, investigating how lipid profiles are altered in AD and their implications for nutrition-based interventions [40, 41]. Finally, metabolic diseases have been reviewed, highlighting the potential of metabolic biomarkers in understanding and treating AD [42].

The study examined the correlation between AD polygenic risk scores and dementia within a UK Biobank cohort. Outcome variables were derived with diagnostic dates obtained through record linkage. The diverse UK Biobank population enabled an objective assessment of exposure-outcome associations. However, limitations were noted. Selection bias and uncertain dementia onset age are significant concerns. Residual confounding may arise from the observational study design, and. reverse causality remains a possibility despite excluding prevalent dementia cases. Structural equation modeling, encompassing GSEM, was employed assuming multivariate normality and no exposure-mediator interactions. Sensitivity analyses confirmed the reliability of the results. Nonetheless, the *med4way* and *gsem* commands cannot fully address exposure-induced mediator-outcome confounding. Healthy volunteer bias and limited generalizability to the broader population are additional concerns. Furthermore, the UK Biobank analysis lacks sufficient power to stratify results by specific racial or ethnic groups.

### Conclusions

In summary, AD PRS is significantly associated with dementia risk, with sex differences and metabolomic pathways providing further insights into dementia etiology. Lipid metabolism, particularly LDL and HDL measures, emerged as potential mediators. This integrative approach highlights the utility of combining genetic and metabolomic data to identify biomarkers and potential targets for early intervention in AD and dementia. Future studies should further examine potential mediators of sex differences in AD PRS-dementia relationships, including through the metabolome. Efforts should also be made to apply these analyses to a more diverse middle-aged adult population and examine pathways explaining racial/ethnic disparities in dementia risk using a multi-omics approach.

## Supplementary Information

Below is the link to the electronic supplementary material.

Supplementary file1 (PDF 262 KB)

Supplementary file2 (XLSX 83.2 KB)

Supplementary file3 (XLSX 56.4 KB)

Supplementary file4 (XLSX 146 KB)

Supplementary file5 (CSV 168 KB)

Supplementary file6 (CSV 60.5 KB)

Supplementary file7 (CSV 161 KB)

## Data Availability

The data are governed by the following licenses and restrictions: The UK Biobank is an extensive biomedical database and research resource that encompasses comprehensive genetic and health data from around 500,000 people in the United Kingdom. The data are routinely enhanced with supplementary records and are universally accessible to authorized researchers doing essential studies on prevalent and life-threatening diseases. Requests for access to these datasets must be submitted to https://www.ukbiobank.ac.uk/.

## Abbreviations

AD: Alzheimer's disease
Aβ: Amyloid beta
CI: Confidence interval
CNS: Central nervous system
CTV3: Clinical Terms Version 3
EHR: Electronic Health Records
ereri_cde: Excess relative risk due to neither mediation nor interaction or controlled direct effect
ereri_intmed: Excess relative risk due to mediated interaction or mediated interaction
ereri_intref: Excess relative risk due to interaction only or reference interaction
ereri_pie: Excess relative risk due to mediation only or pure indirect effect
GlycA: Glycoprotein acetyls
GP: General Practice
GPC: Genetic Principal Component
GWAS: Genome-wide association studies
HES: Hospital Episode Statistics
HR: Hazard ratio
ICD-10: International Classification of Diseases, Tenth Revision
LDL: Low-Density Lipoproteins
MRI: Magnetic resonance imaging
OLS: Ordinary lease squares
PCA: Principal Components Analysis
pct_cde: Percent of total effect that is controlled direct effect
pct_intmed: Percent of total effect that is mediated interaction
pct_intref: Percent of total effect that is reference interaction
pct_pie: Percent of total effect that is pure indirect effect
PCA: Principal Components Analysis
PC: Principal Component
PH: Proportional hazards
PIE: Pure Indirect Effects
PRS: Polygenic Risk Score
Ref: Referent category
SD: Standard Deviation
TDI: Townsend Deprivation Index
tereri: Total excess relative risk
UK: United Kingdom

## Code availability

All codes and related output, datasheets and Figures will be made available on github at https://github.com/baydounm/UKB-paper21-supplementarydata.
