# Supplementary material for: Alzheimer’s disease polygenic risk’s association with all-cause dementia through the plasma metabolome in the UK Biobank study
Source: GeroScience. 2025 Jul 1;47(6):7023–42. doi: 10.1007/s11357-025-01724-4 (PMC12638481; doi:10.1007/s11357-025-01724-4)
Supplement: Supplementary file 1 — Supplementary file1 (PDF 262 KB) [file 11357_2025_1724_MOESM1_ESM.pdf]

ONLINE SUPPLEMENTARY MATERIALS

OSM 1: AD Polygenic risk scores and genetic principal components

The study focuses on the development and application of PRS scores to meta-analyzed GWAS summary statistics, either extracted from external GWAS data or a combination of external and internal UK Biobank data. The Standard PRS Set, which includes 28 diseases and 8 quantitative traits, was generated using external GWAS data. The International Genomics of Alzheimer's Project (IGAP) is an extensive three-stage study based on genome-wide association studies (GWAS) conducted on people with European ancestry.

The 2022 updated AD PRS pipeline outlines the essential steps in this process: selecting appropriate polygenic risk scores from the literature, extracting pertinent genetic variants and confirming their quality, calculating the risk score, and important considerations for its inclusion in statistical models. The tutorial highlights several special methods necessary when utilizing data in the forms that UK Biobank has chosen, such as dealing with large numbers of variants and evaluating the suitability of tools.

The European Alzheimer & Dementia Biobank (EADB) consortium has compiled a dataset of 20,464 clinically diagnosed AD cases and 22,244 controls from 15 European countries. The results were meta-analyzed using a proxy-AD GWAS from the UK Biobank dataset. A genome-wide association study involving 111,326 AD cases and 677,663 controls identified 75 risk loci, 42 of which were new. A new genetic risk score was created, resulting in a 1.6- to 1.9-fold increase in AD risk, adjusting for age and the APOE e4 allele.

The secondary analysis in this paper was carried out on the 2022 AD PRS, mainly to contrast its association with metabolome with the main AD PRS that was found in a previous study to be associated with ApoE4 status, where more details on AD PRS construction is provided(1).

Sources:

https://www.medrxiv.org/content/10.1101/2022.06.16.22276246v1.supplementary-material?versioned=true

https://www.pgscatalog.org

(1, 2, 3, 4, 5)

OSM 2: Other covariates

Age was documented at baseline and is a crucial factor influencing health outcomes and neurodegenerative mechanisms. Sex was classified as male or female, while race/ethnicity was self-reported and grouped into primary groups. Education level was self-reported and classified into achievement categories, acting as a surrogate for cognitive reserve and socioeconomic status. Income was self-reported and utilized as an ordinal variable, indicating financial resources that may influence access to healthcare, lifestyle, and environmental exposures. Household size was defined as the total number of individuals residing in the same household as the participant. Education, income and Townsend deprivation index measured at baseline assessment were combined into a single average z-score when at least two of the three indicators were non-missing. This resulted in an SES score which was used as a single summary measure in all models along with age, sex, age-squared, genetic principal components and household size. Description of the Townsend deprivation index, a measure of area-level deprivation at the postcode level, along with education and income categorization, is detailed elsewhere(6, 7).

Source: https://biobank.ndph.ox.ac.uk/showcase/

OSM 3: Principal components analysis

Principal Components Analysis (PCA) is a method to reduce a dataset's dimensionality by dividing 249 metabolites into 15 principal components. This involves eigen decomposition, computation of main component scores, variance attribution, and variance optimization. Varimax rotation is applied to optimize squared loadings so as to obtain a simple structure whereby each component has distinctive patterns of measured variables with elevated component loadings. The predicted components using the regression method are subsequently normalized and converted to a z-score. This procedure is performed using Stata commands.

Source: (8)

(Eq. 1.1) *Calculate covariance (or correlation matrix)*: $\sum = \boldsymbol{\frac{1}{n-1} M^T M}$

(Eq. 1.2) *Eigenvalue decomposition*: $\sum v_k = \lambda_k v_k$

(Eq. 1.3) *Compute principal components scores*: $PC_{ik} = \sum_{j=1}^{249} M_{ij} v_{jk}$

(Eq. 1.4) Varimax rotation of loadings: $L = VT$

(Eq. 1.5) *Prediction equation after rotation*: $\boldsymbol{PC'_{ik} = \sum_{j=1}^{249} M_{ij} l_{jk}}$

(Eq. 1.6) *Standardize the predicted rotated PC*: $PC''_{ik} = \frac{PC'_{ik} - \mu_k}{\sigma_k}$

OSM 4: Generalized structural equations modeling

Generalized Structural Equation Modeling (GSEM) in Stata is a flexible framework that estimates both linear and non-linear connections among variables, dependent variables, and random effects. It accommodates various distributions and link functions, employing Maximum Likelihood or Quasi-ML methodologies. Goodness-of-Fit statistics and tests evaluate model adequacy, facilitating linear prediction, residual estimates, and indirect effects assessment.

Here, we estimated a generalized structural equation model incorporating the following equation :

- A Weibull model (e.g., hazard function for dementia incidence).
- A main exposure (X) for AD PRS at the individual level.
- A main mediator (M) of metabolomic marker at the individual level.
- Exogenous variables (Z_i) at individual level.

1. Outcome Equation (Weibull Model)

The survival time (T) for each transition j is model as:

(Eq. 2.1) $h_{ij}(t) = k\lambda_{ij}^{k_{ij}} t^{k-1}$

Where:

$h_{ijk}(t)$: Hazard function for individual i for transition j and local authority k at time t.

k: Shape parameter of the Weibull distribution (assumes proportional hazards if constant)

λ_{ij}: Scale parameter for individual i, transition j, modeled as:

(Eq. 2.2) $\ln(\lambda_{ij}) = \beta_{0j} + \beta_{1j}X_i + \beta_{2j}M_i + \sum_{p=1}^{P} \beta_{3jp} Z_{p,i}$

X_i: AD PRS (main or newly derived) for individual i.

M_i: Potentially mediating metabolomic marker for individual i.

$Z_{p,i}$: Exogenous covariate p for individual i.

2. Mediator Equation

The potential mediator, each alternative metabolomic marker, is modeled as follows:

(Eq. 2.3) $\mathrm{M}_i = \gamma_{0j} + \gamma_{1j}X_i + \sum_{p=1}^{P} \gamma_{3jp} Z_{p,i}$

X_i: AD PRS (main or newly derived) for individual i.

M_i: Potentially mediating metabolomic marker for individual i.

$Z_{p,i}$: Exogenous covariate p for individual i.

3. Exposure Equation

The main exposure, AD PRS, is modeled as follows:

(Eq. 2.4) $X_i = \alpha_{0j} + \sum_{p=1}^{P} \alpha_{jp} Z_{p,i}$

X_i: AD PRS (main or newly derived) for individual i.

$Z_{p,i}$: Exogenous covariate p for individual i.

Source: (9)

OSM 5: Four-way decomposition models

Stata introduced the command med4way which can be freely obtained on github at https://github.com/anddis/med4way, using methods described by Discacciati et al. (2018)(10). This Stata command is intended for the analysis of mediation and interaction effects utilizing the four-way effect decomposition methodology(10). This technique disaggregates the overall impact of an exposure on an outcome into four separate component

Controlled Direct Effect (CDE): The direct impact of the exposure on the outcome, while maintaining the mediator at a constant level.

Pure Indirect Effect (PIE): The influence of the exposure on the outcome that functions solely through the mediator.

Reference Interaction (INTref): The segment of the impact ascribed to the interaction between the exposure and mediator when the mediator is maintained at its reference value.

Mediated Interaction (INTmed): The portion of the effect resulting from the interaction between the exposure and the mediator when the mediator varies.

This deconstruction enables researchers to evaluate the relationship between mediation and interaction processes thoroughly(10). Discacciati and colleagues discuss the utility of *med4way* for investigating causal processes and its applicability in epidemiological research to elucidate complex interactions among variables(10).

Supplementary References

1. Beydoun MA, Beydoun HA, Li Z, Hu YH, Noren Hooten N, Ding J, et al. Alzheimer's Disease polygenic risk, the plasma proteome, and dementia incidence among UK older adults. Geroscience. 2024.
2. Kunkle BW, Grenier-Boley B, Sims R, Bis JC, Damotte V, Naj AC, et al. Genetic meta-analysis of diagnosed Alzheimer's disease identifies new risk loci and implicates Abeta, tau, immunity and lipid processing. Nat Genet. 2019;51(3):414-30.
3. Collister JA, Liu X, Clifton L. Calculating Polygenic Risk Scores (PRS) in UK Biobank: A Practical Guide for Epidemiologists. Front Genet. 2022;13:818574.
4. Bellenguez C, Kucukali F, Jansen IE, Kleineidam L, Moreno-Grau S, Amin N, et al. New insights into the genetic etiology of Alzheimer's disease and related dementias. Nat Genet. 2022;54(4):412-36.
5. Bycroft C, Freeman C, Petkova D, Band G, Elliott LT, Sharp K, et al. The UK Biobank resource with deep phenotyping and genomic data. Nature. 2018;562(7726):203-9.
6. Beydoun MA, Beydoun HA, Hu YH, Li Z, Georgescu MF, Noren Hooten N, et al. Mediating and moderating effects of plasma proteomic biomarkers on the association between poor oral health problems and brain white matter microstructural integrity: the UK Biobank study. Mol Psychiatry. 2024.
7. Beydoun MA, Beydoun HA, Noren Hooten N, Meirelles O, Li Z, El-Hajj ZW, et al. Hospital-treated prevalent infections, the plasma proteome and incident dementia among UK older adults. iScience. 2023;26(12):108526.
8. Rabe-Hesketh S, Everitt, B. S., . A Handbook of Statistical Analyses Using Stata, 4th edition. : Chapman & Hall/CRC Press.; 2004.
9. Acock A. C. Structural Equation Modeling with Stata (revised edition). College Station, TX Stata Press; 2013.
10. Discacciati A, Bellavia A, Lee JJ, Mazumdar M, Valeri L. Med4way: a Stata command to investigate mediating and interactive mechanisms using the four-way effect decomposition. Int J Epidemiol. 2018.
