# Supplementary figures and images for: Alzheimer’s disease polygenic risk’s association with all-cause dementia through the plasma metabolome in the UK Biobank study
Source: GeroScience. 2025 Jul 1;47(6):7023–42. doi: 10.1007/s11357-025-01724-4 (PMC12638481; doi:10.1007/s11357-025-01724-4)

Supplementary Figure 1. Participant Flowchart

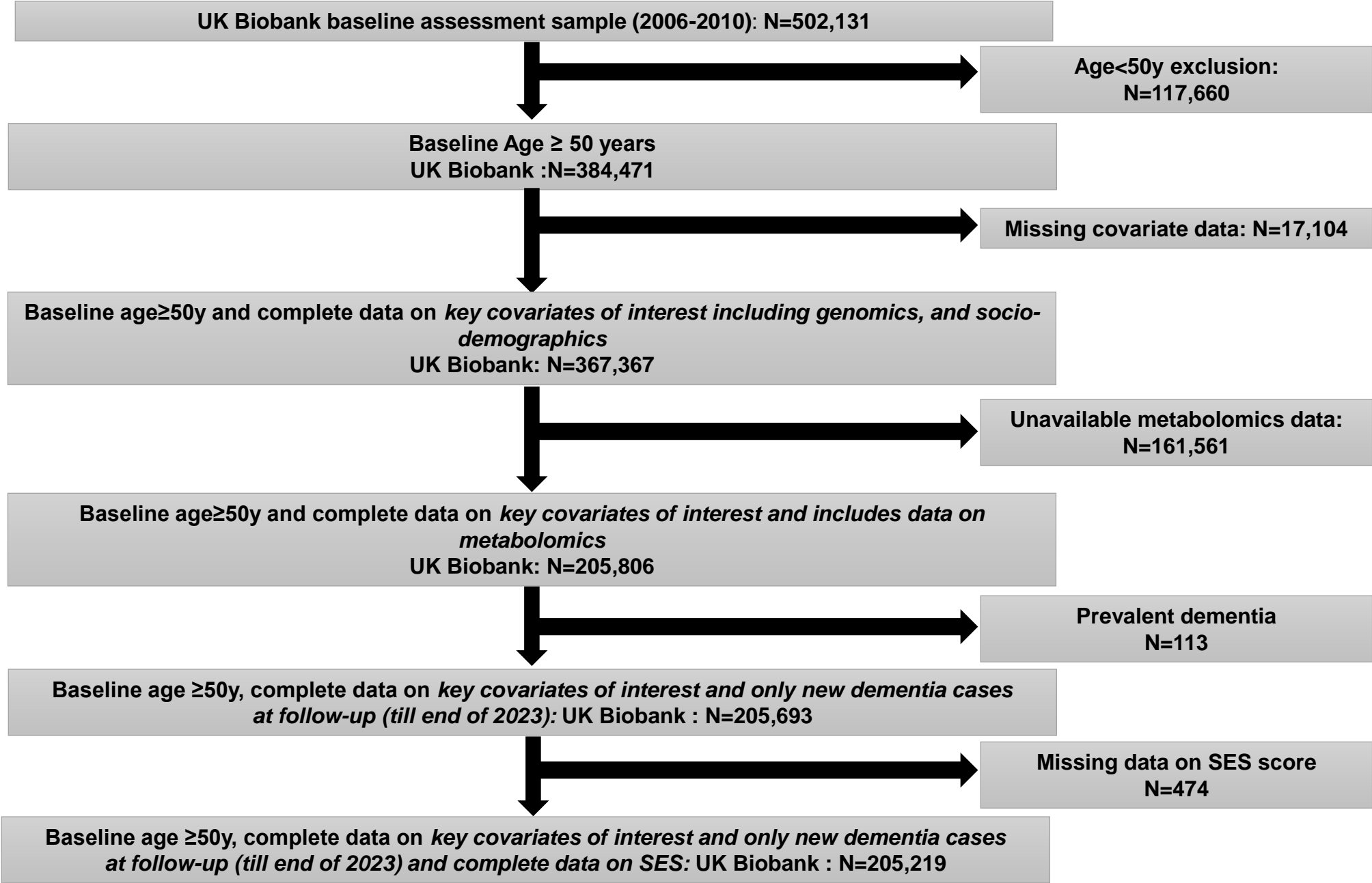

Supplement: Supplementary file 6 — Supplementary file6 (CSV 60.5 KB) [file 11357_2025_1724_MOESM6_ESM.pdf]
