# Supplementary material for: Alzheimer’s disease polygenic risk’s association with all-cause dementia through the plasma metabolome in the UK Biobank study
Source: GeroScience. 2025 Jul 1;47(6):7023–42. doi: 10.1007/s11357-025-01724-4 (PMC12638481; doi:10.1007/s11357-025-01724-4)

FIGURE S2. Findings from four-way decomposition models for main AD PRS vs. dementia incidence through 15 metabolomic prinicipal components (z-scored): Pure indirect effects across 10 random sub-samples and pooled effect: UK Biobank Study 2006-2023

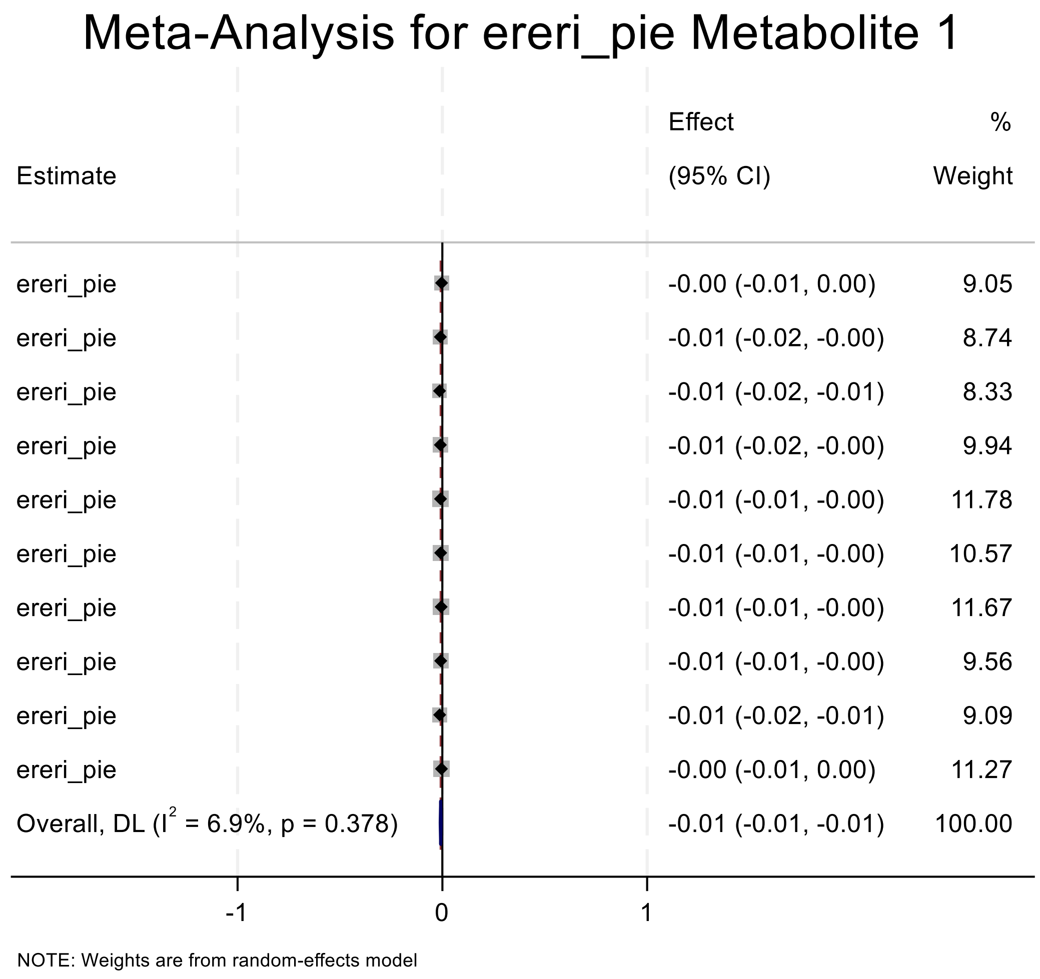

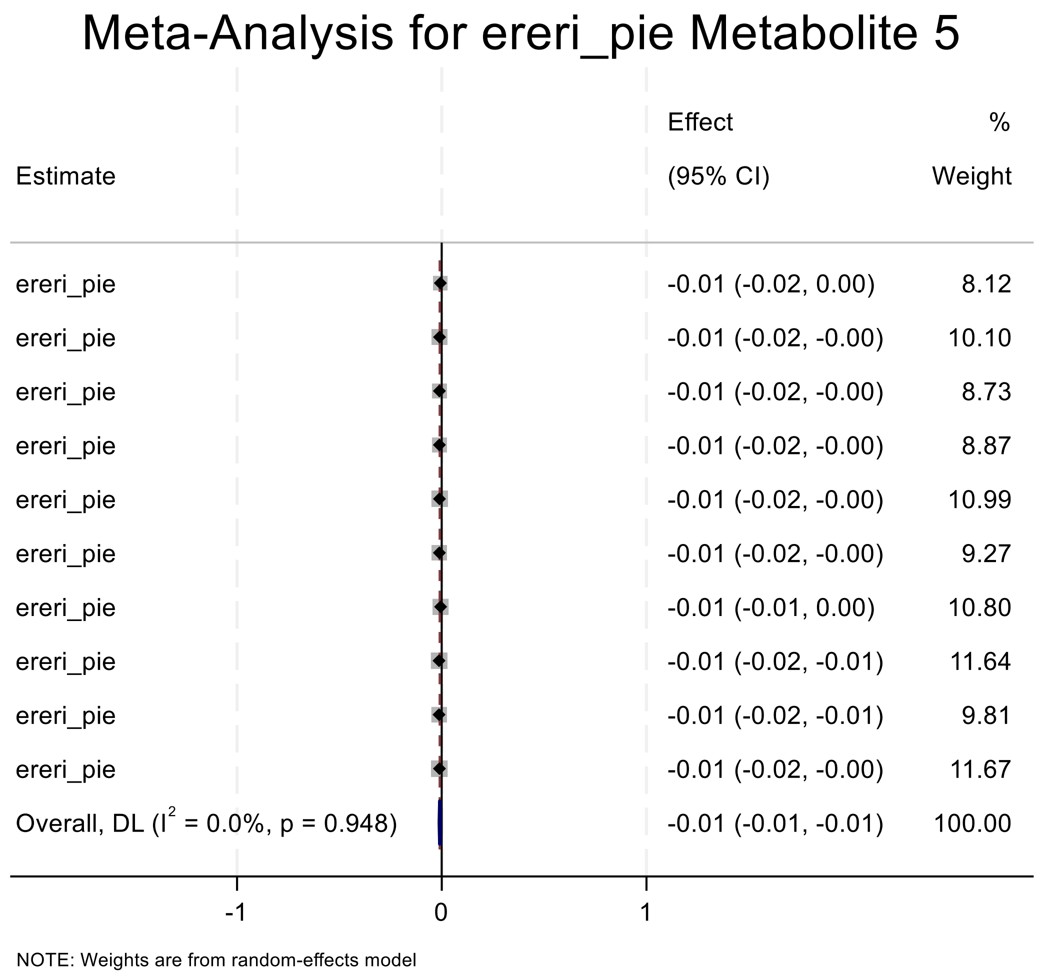

Supplement: Supplementary file 7 — Supplementary file7 (CSV 161 KB) [file 11357_2025_1724_MOESM7_ESM.pdf]
